# Unintended consequences of the physical and health integration policy in China’s primary healthcare: a grounded theory study of policy implementation dynamics

**DOI:** 10.3389/fpubh.2025.1707846

**Published:** 2025-12-15

**Authors:** Weining Yang, Xuetao Jiang, Xue Luo, Xi Zhang, Lu Yan

**Affiliations:** 1 Institute of Physical Education, Kunming University of Science and Technology, Kunming, China; 2 Research Institute of Higher Education, Yunnan University, Kunming, China

**Keywords:** physical and health integration, primary healthcare, policy implementation, unintended consequences, grounded theory

## Abstract

**Introduction:**

Under the framework of the “Healthy China” initiative, the Physical and Health Integration (PHI) policy has become a strategic tool to promote health-oriented transitions in primary healthcare. While existing research has largely focused on policy design and expected outcomes, limited attention has been devoted to the actual implementation processes and unintended consequences at the frontline. This study addresses this gap by examining how frontline practitioners enact, adapt, and reinterpret the PHI policy in practice.

**Methods:**

This study employs grounded theory to explore the dynamic mechanisms shaping PHI policy implementation. Drawing on 20 in-depth interviews with primary healthcare practitioners across China, unintended consequences were identified through open, axial, and selective coding. The analytical process enabled the extraction of recurrent patterns and the development of a mechanism-based explanatory model.

**Results:**

The analysis reveals a non-linear, chain-like implementation process characterized by cognitive misalignment, resource and institutional constraints, and diversified behavioral strategies among frontline actors. Unintended consequences emerged through symbolic compliance, resource distortion, and behavioral adaptation. Frontline practitioners adopted varied strategies—from passive compliance and symbolic fulfillment to localized adaptation—resulting in fragmented implementation. These interacting mechanisms formed a self-reinforcing cycle that reproduced gaps between policy intentions and practice.

**Discussion:**

The findings align with broader policy implementation theories. Symbolic compliance reflects Lipsky’s Street-Level Bureaucracy perspective, wherein frontline actors adapt or distort directives to accommodate local constraints. Similarly, Pressman and Wildavsky’s Implementation Gap framework helps explain how limited resources and ambiguous guidance contribute to deviations between intended and actual policy outcomes. Based on these insights, the study proposes a “mechanism model of unintended consequences” that highlights structural challenges related to intersectoral coordination, knowledge translation, and performance incentives. The study contributes to public policy implementation research by offering a micro-level explanation of how ambitious health reforms are translated, negotiated, and reframed in grassroots contexts.

## 1 Introduction

Physical exercise has long been recognized in China as a vital contributor to health. Over two thousand years ago, the classical medical text *Huangdi Neijing* proposed the concept of “treating disease before it arises,” advocating movement and breathing techniques to strengthen the body and prevent illness (1). While the idea that “exercise promotes health” is widely accepted, exercise-based interventions were not formally incorporated into China’s health agenda until the National Fitness Program of 2002. The *Healthy China 2030 Planning Outline*, launched in 2016, signaled a further shift, with President Xi Jinping calling for the integration of nationwide fitness and public health. Against this backdrop, the PHI strategy was incorporated into China’s national health governance system as a pivotal mechanism for advancing preventive care and restructuring the public health framework (2).

The PHI strategy integrates physical activity resources with medical and health systems, establishing exercise-based interventions as a core means of influencing behavior. It seeks to link individual practices, community services, and institutional support to address chronic disease, population aging, and fragmented health services, while also enhancing the overall population health (3). This approach aligns with the globally recognized principle of Exercise is Medicine (4).

Compared with similar policies in Western countries, China’s PHI strategy differs in both implementation pathways and institutional safeguards. In the United States, the Exercise is Medicine initiative has been promoted since the 1980s through cross-sector collaboration led by the American College of Sports Medicine (ACSM) and the Centers for Disease Control and Prevention (CDC), forming a nationwide network of exercise-based intervention services (5). In the United Kingdom, the National Quality Assurance Framework for Exercise Referral Systems institutionalizes interdisciplinary collaboration, emphasizing the roles of primary healthcare institutions, local governments, and community sports organizations (6). Japan’s community care system, which integrates healthcare, welfare, and community services, also offers valuable insights for improving local-level coordination and enhancing the sustainability of health-promoting programs. Likewise, Finland’s health-promoting hospitals model, which fosters collaboration across sectors and emphasizes the role of healthcare facilities in supporting community health, provides a successful example of integrated policy implementation. By contrast, China’s strategy has been driven mainly by top-down government directives, with limited interdepartmental coordination and insufficient local-level innovation (7). Drawing lessons from these international models could offer important strategies for strengthening China’s PHI policy, particularly in terms of fostering cross-sector collaboration and improving long-term sustainability at the grassroots level.

Within China’s health governance system, primary healthcare institutions play a pivotal role at the intersection of public health, sports services, and medical care. They are responsible for delivering exercise interventions, health counseling, and chronic disease management to improve community outcomes (8). While previous research has explored the policy design and public health value of PHI (8, 9), less is known about its unintended consequences at the primary healthcare level. In particular, limited attention has been paid to how frontline medical staff interpret, respond to, and emotionally navigate the policy in practice. As a result, two key questions remain insufficiently addressed:

- How do primary healthcare workers perceive and understand the PHI policy?
- Why do their actual behavioral responses deviate from policymakers’ intended expectations?

This study aims to examine the unintended consequences of implementing the PHI policy in China’s primary healthcare system. Using a Grounded Theory approach, it explores the mechanisms underlying these effects from the perspective of grassroots healthcare practitioners. By introducing a comparative international lens, the study not only enriches empirical evidence on China’s PHI practice but also offers a novel explanatory framework for global health governance discourse (10). Specifically, the mechanism model developed in this research reveals how cognitive deviations, institutional fragmentation, and adaptive behaviors jointly produce unintended consequences, providing a micro-level explanation for the policy–practice gap.

## 2 Literature review

### 2.1 Policy framework and objectives of physical and health integration

PHI is a collaborative model linking the sports and healthcare systems, focused on disease prevention, treatment, and rehabilitation. Its core objective is to improve public health through systematic exercise interventions, thereby transforming individual behavior from passive reception to proactive engagement (9). Central to this approach are exercise prescriptions, health guidance, and the integration of medical services to achieve population-wide health improvement (2).

Globally, PHI, alongside the Exercise is Medicine (EIM) initiative, has emerged as a key strategy for addressing chronic disease and public health challenges. Since the 1980s, the United States has developed a cross-sectoral system led by the American College of Sports Medicine (ACSM) and the Centers for Disease Control and Prevention (CDC), embedding exercise interventions into clinical pathways and facilitating collaboration among physicians, exercise specialists, and community resources (5). Similarly, the United Kingdom promotes an “exercise referral” model under the National Quality Assurance Framework, emphasizing interdisciplinary teamwork, community involvement, and local government support (6).

Since the release of the Healthy China 2030 Planning Outline, PHI has become a national strategic priority in China, emphasizing the role of primary healthcare institutions in delivering exercise prescriptions, health management, and chronic disease prevention. However, compared with Western countries, China’s approach faces limitations in inter-departmental resource integration, local government autonomy, and professional training for frontline medical staff (7). These gaps highlight the need to examine the unintended consequences of policy implementation at the grassroots level.

### 2.2 The role and challenges of primary healthcare

Community health centers and township hospitals serve as central nodes, providing clinical services while promoting health and managing chronic diseases (8, 11). However, implementing PHI policies at the primary level faces multiple challenges. Many grassroots institutions remain focused on clinical treatment, with limited investment in health promotion and exercise-based interventions (12). Frontline healthcare workers often lack systematic training in exercise medicine and health management, hindering integration of medical and sports resources (13). Additionally, under a predominantly top-down policy model, primary healthcare staff frequently occupy a passive role, with insufficient incentives and institutional support, reducing the effectiveness of policy implementation.

In contrast, research in global contexts emphasizes frontline workers’ autonomy and agency. According to Street-Level Bureaucracy theory, implementers make strategic choices based on experience, interests, and contextual constraints, particularly under resource limitations and conflicting demands (14). Understanding how primary healthcare staff interprets and responds to PHI policies is therefore essential for evaluating policy effectiveness.

### 2.3 Unintended consequences

Unintended consequences refer to outcomes of policy implementation that deviate—positively or negatively—from the original goals (15). While public policy studies, unintended consequences are acknowledged as having both beneficial and adverse effects, current research largely emphasizes macro-level objectives, with limited focus on frontline healthcare staff’s behavioral responses (10). Although international studies have explored institutional innovation and cross-sector collaboration in PHI, few have addressed the cognitive conflicts, behavioral adaptations, and emotional responses of Chinese grassroots health workers. Similarly, Chinese literature has largely concentrated on policy design and outcome evaluation, with limited research on implementation processes and the mechanisms behind unintended effects.

### 2.4 Research gap

Despite growing interest in PHI as a core component of China’s national health strategy, existing research mainly addresses three dimensions:

- Policy design and institutional frameworks, exploring top-down structures and governance logics (7, 9);
- Macro-level comparisons of policy objectives and practices, particularly differences and adaptations between China and Western countries (2, 6);
- Policy effectiveness and health outcomes, evaluating measurable changes in health indicators and system responses (4).

However, three key gaps remain underexplored:

- Lack of micro-level implementation perspectives: Most studies focus on policy design or system-level analysis, paying limited attention to how primary healthcare practitioners experience, interpret, and respond to policies. This gap hinders understanding of the challenges in translating top-level directives into frontline practice.
- Insufficient theorization of unintended consequences: Although interest in policy deviations during implementation is growing, existing research lacks a systematic framework for understanding the mechanisms through which they arise, particularly how cognitive, resource-based, and institutional factors interact to shape divergent behaviors and outcomes.
- Weak integration of local evidence with international frameworks: Although PHI in China operates within a unique institutional and cultural context, few studies effectively link local implementation logics with internationally recognized theories of policy execution. Greater “localized translation” of global conceptual tools is needed.

Existing policy implementation theories generally recognize that policy deviation is an inherent feature of complex governance rather than a simple failure of execution. From the perspective of street-level bureaucracy, frontline actors reinterpret and adjust policy goals according to their discretion, workload, and resource constraints. The implementation gap model further indicates that deviations accumulate as abstract policy directives are translated through multiple administrative layers, while multi-level governance theory emphasizes that fragmentation and interdependence across sectors can intensify these divergences. In this sense, deviation represents both an adaptive mechanism and a constraining force, shaping the ways policies materialize in real-world contexts.

To empirically address these dynamics, this study employs Grounded Theory to inductively uncover the mechanisms through which policy deviation occurs. By deriving categories such as cognitive misalignment, resource constraint, and strategic adaptation directly from frontline experiences, Grounded Theory bridges the gap between theoretical expectations of deviation and the observed realities of policy implementation, thereby grounding abstract assumptions in practical evidence.

In response, this study focuses on the context of China’s primary healthcare system, drawing upon international theoretical frameworks to address the above gaps. It proposes a new analytical perspective—a causal chain linking policy design→cognitive interpretation→behavioral response→unintended consequences to offer both theoretical insight and empirical evidence for improving policy implementation in complex governance environments.

## 3 Research design

### 3.1 Research methodology

Grounded Theory, a classical qualitative methodology, enables researchers to extract core concepts from empirical data and construct theoretical frameworks by analyzing their interrelationships (16). It is particularly valuable for discovering and interpreting new phenomena, as it enables iterative refinement of theoretical assumptions throughout extended data collection and analysis. Consequently, Grounded Theory has become one of the most influential and widely applied methods in the social sciences (17).

This study employs Grounded Theory as the primary methodology to investigate the unintended consequences arising during the implementation of the PHI policy from the perspective of primary healthcare practitioners. By leveraging data-driven, inductive techniques, the study aims to uncover the underlying mechanisms shaping policy implementation outcomes (16, 17).

### 3.2 Data collection

This study adopted purposeful sampling to recruit 20 primary healthcare practitioners from three regions in China: East, Central, and Southwest. Specifically, 8 participants were from the East, 5 from the Central region, and 7 from the Southwest region. Among them, 12 participants worked in community health centers and 8 were employed in township health institutions. All participants had at least two years of frontline experience and were directly involved in the implementation of the PHI policy. In terms of demographics, 9 participants were male and 11 were female. Their years of professional experience ranged from 3 to 32 years, with an average of 6.8 years. The inclusion of participants from diverse institutional backgrounds and regions was intended to capture variations in policy implementation and to generate a more comprehensive understanding of the PHI policy at the grassroots level.

During data collection, the researchers developed an interview protocol of 10 open-ended questions, informed by the literature review and theoretical framework. The questions explored participants’ interpretations of the policy, its impacts on behavior and workflow, and their suggestions for improvement. Data were collected through semi-structured interviews lasting 50–60 min each, all of which were audio-recorded and transcribed verbatim within 24 h. In total, over 1,200 min of audio were collected, yielding approximately 160,000 words of qualitative data.

### 3.3 Coding process overview

Following data collection, the qualitative materials were analyzed using a three-stage iterative process, including open, axial, and selective coding, aligned with Grounded Theory principles.

The open coding stage involved examining each transcript line by line to generate initial concepts that captured participants’ interpretations and lived experiences of PHI. The coding was conducted by two researchers who independently coded the data. Any discrepancies between the coders were discussed and resolved through consensus. In addition, triangulation was employed by incorporating multiple data sources to ensure the comprehensiveness and credibility of the findings.

The axial coding stage connected these subcategories by identifying relationships across cognitive, resource, and institutional dimensions, forming broader categories that explained implementation dynamics.

Finally, selective coding integrated these categories into a coherent theoretical structure that linked policy cognition, contextual constraints, behavioral strategies, and unintended consequences. This sequential process ensured that theory emerged directly from the empirical data in a transparent and reproducible manner.

### 3.4 Open coding: refining concepts and scope

Open coding involves line-by-line analysis of raw data to generate initial concepts, assign labels, and group them into emergent categories (18). To minimize bias, researchers remained free from preconceptions and avoided imposing subjective interpretations. Following this principle, NVivo software was used to analyze the verbatim transcripts, enabling systematic identification of concepts that reflected key patterns and phenomena in policy implementation.

The open coding process emphasized preserving the original semantics of participants’ responses to maintain close alignment with the data (19). Through iterative line-by-line annotation and constant comparison, 158 initial concepts were identified and subsequently refined into 17 subcategories. Table 1 presents a partial overview of this process (only a selection is shown due to space constraints).

**Table 1:** Open coding (partial examples).

Sub-category	Initial concept	Original statement
A1 Misinterpretation of policy objectives	G1 Confusion between health promotion and disease treatment.	“Doctors focus solely on disease treatment. Prevention is mostly up to the patient. Following medical advice is considered the best form of health promotion.”
	G2 Excessive emphasis on short-term outcomes.	“Hospitals pursue quick symptom relief. If there’s no short-term effect, patients will not even come—let alone think about long-term health.”
E1 Enrichment of doctor-patient communication channels	G53 Increased interaction time with patients	“We now spend much more time communicating with patients about daily monitoring and physical activity.”
	G54 Expanded communication content.	“Some patients visit specifically to give feedback on their exercise outcomes and to discuss the next steps with me.”

### 3.5 Axial coding

Axial coding aims to develop the properties and dimensions of initial categories and to identify potential logical relationships among them. By continuously comparing and connecting initial concepts, this phase generates intermediate-level categories that explain key issues in policy implementation.

This study refined 17 subcategories from the open coding phase and grouped them into five overarching categories (core themes). These categories correspond to three intermediary pathways—Cognitive Pathway (Z1), Resource Pathway (Z2), and Institutional Pathway (Z3)—as well as Behavioral Strategies (Z4) and Unintended Consequences (Z5). Table 2 provides the full results of axial coding.

**Table 2:** Axial coding.

Core category	Sub-category	Category description
Z1 Cognitive pathway	A1 Misinterpretation of policy objectives	Primary healthcare personnel fail to accurately grasp the goals of physical and health integration, leading to misalignment.
	A2 Ambiguity of exercise intervention concept	Limited understanding of “exercise intervention” and lack of comprehensive knowledge in health promotion.
	A3 Insufficient interdisciplinary collaboration awareness	Inadequate mutual recognition between healthcare and sports sectors hinders interdisciplinary cooperation.
	A4 Oversimplification of implementation process	Implementation is overly simplified, neglecting the complexity of cross-sectoral coordination and operational details.
Z2 Resource pathway	B1 Insufficient financial support	Inadequate funding limits key implementation components such as space, equipment, and human resources.
	B2 Shortage of professionals	Lack of experts in sports medicine and health management results in personnel shortages in PHI execution.
	B3 Lack of specialized equipment	Absence of essential tools like exercise monitoring devices and rehabilitation equipment undermines policy outcomes.
	B4 Difficulties in interdepartmental coordination	Cross-departmental collaboration is hindered by unclear responsibilities, resource fragmentation, and poor coordination.
Z3 Institutional pathway	C1 Mismatch of workflows	Existing service models and workflows are misaligned with the structural changes required by PHI policy.
	C2 System integration barriers	Communication and coordination between healthcare and sports departments are ineffective, reducing policy efficiency.
	C3 Lack of professional training	Lack of training in sports medicine and health management constrains integration of healthcare and sports services.
	C4 Performance evaluation challenges	Absence of effective performance metrics and evaluation mechanisms impairs policy feedback and accountability.
Z4 Behavioral strategies	D1 Passive execution	Execution is minimal and reactive due to limited awareness and resources.
	D2 Formal compliance	Symbolic adherence occurs under pressure, without substantive intervention.
	D3 Strategic adaptation	Organizations with sufficient knowledge and support initiate innovative and localized execution strategies.
Z5 Unintended consequences	E1 Positive unintended consequences	Improved doctor-patient communication, enhanced learning, and increased awareness of physical health.
	E2 Negative unintended consequences	Fragmented execution, resource waste, and implementation fatigue due to policy design flaws and misaligned execution.

### 3.6 Selective coding

Integrating open and axial coding, the study constructs a storyline of PHI policy implementation in China’s primary healthcare system. Although the policy aims to enhance chronic disease management through collaboration between sports and healthcare systems, its top-down dissemination—marked by abstract language and cross-sector complexity—has produced fragmented interpretations among frontline implementers.

From the early stages of rollout, diverse interpretations have produced cognitive deviations along the Cognitive Pathway (Z1). As implementation advances, actors face dual constraints from the Resource Pathway (Z2)—including personnel, funding, and equipment shortages—and the Institutional Pathway (Z3), characterized by limited coordination between the sports and health sectors. Furthermore, fragmented systems for performance evaluation, data interoperability, and service workflows further hinder integrated implementation.

In response to these constraints, frontline implementers adopt divergent behavioral strategies (Z4). Some institutions proactively pursue adaptive innovations tailored to local needs, whereas many default to passive compliance or formalistic responses aimed at meeting surface-level performance metrics. The distinction between “formal compliance” and “strategic adaptation” is important to highlight. “Formal compliance” refers to instances in which frontline actors follow prescribed procedures or policy requirements without making substantive changes, often to preserve appearances or satisfy performance evaluations. For example, one healthcare worker remarked, “We complete the forms and hold the exercise events, but there’s little real follow-up or assessment” (FTDX_15). In contrast, “strategic adaptation” involves modifying implementation processes to better suit local contexts, even when such adjustments deviate from official guidelines. As one community health center director noted, “We tailored the program to meet the specific needs of our older population, even though it wasn’t strictly in the guidelines” (FTDX_04). These strategies produce varied policy outcomes, and over time, multiple unintended consequences (Z5) have emerged. Although a few innovative cases foster interdepartmental cooperation and patient engagement, most implementations remain superficial or symbolic, leading to policy fatigue, resource waste, and declining institutional trust. Delayed feedback on policy effectiveness often conceals these issues, causing subsequent policies to reproduce the same flawed patterns.

**Figure 1:**
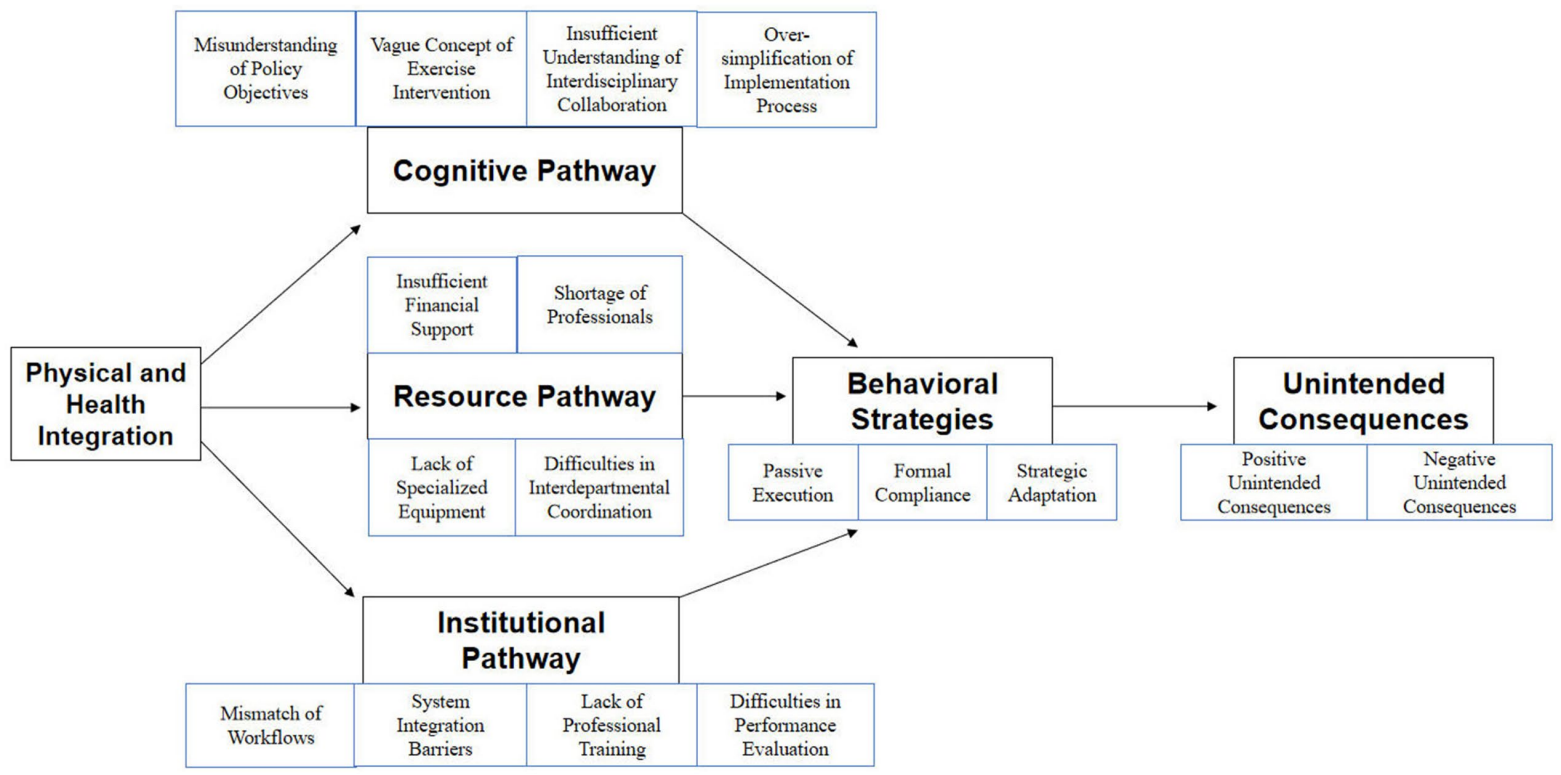
Mechanism model of unintended consequences in the implementation of physical and health integration policy.

Figure 1 displays these dynamics. Mechanism Model of Unintended Consequences in the Implementation of PHI Policy.

### 3.7 Theoretical saturation test

After coding 16 interview transcripts, analysis of the subsequent three yielded no new major categories or interrelationships among the existing categories. The conceptual framework remained stable, confirming its theoretical foundation.

## 4 Model explanation

### 4.1 Interpretation of policy cognition: the reconstruction and alienation of policy discourse at the grassroots level

Policy cognition determines how implementers interpret goals, understand key concepts, and develop action strategies. Policy implementation research increasingly emphasizes that policy is not linear but is “re-interpreted” and “reconstructed” within specific contexts, producing differentiated outcomes (20). In China, policy discourse is often highly abstract and politically oriented. During grassroots dissemination and localization, it frequently experiences goal ambiguity, semantic drift, and practical alienation (21, 22). This study finds that thePHI policy, embedded within this complex discursive environment, has undergone multiple layers of cognitive transformation, resulting in divergent implementation paths.

First, ambiguity in policy objectives has led frontline actors to misinterpret the policy’s original intentions (A1). Although the texts emphasize improving chronic disease management through integration of sports and healthcare, many primary care physicians view the policy primarily as an administrative task imposed by higher authorities, neglecting its service-oriented transformation agenda. As one community hospital director explained: “They call it ‘integration,’ but in practice, it just means organizing an event, running a health check, and making sure the numbers look good in the report” (FTDX_16). This illustrates administrative recoding within the implementation system, where practitioners prioritize accountability to superiors over internalizing the policy’s substantive goals (23).

Second, the term “exercise intervention” is interpreted inconsistently across sectors, revealing the conceptual ambiguity common in cross-disciplinary policies (A2). Some physicians reduce it to simply advising patients to “exercise more,” while sports administrators treat it as an extension of fitness programming—neither aligning with the policy’s intended role as a personalized, prescription-based intervention. This lack of clarity impedes standardization and blurs professional responsibilities. As one physician candidly noted: “Intervention? That just means telling them to move more. Writing an actual exercise prescription? Honestly, we do not really know how to do that” (FTDX_03). In public health, blurred knowledge boundaries are recognized as obstacles to effective cross-sector collaboration (24).

Although the policy promotes interdepartmental cooperation, the sports and healthcare systems lack a shared understanding or operational platform (A3). Both sides demonstrate limited clarity about roles and boundaries, rendering “integration” a little more than a mechanical juxtaposition of segmented departmental functions. This cognitive rupture reflects entrenched path dependency within bureaucratic systems, reinforcing the reactive role of institutional boundaries in shaping professional cognition (25).

Finally, in practice, implementation has been reduced to a three-step model—“recording—informing—rechecking” (A4)—with a little attention to process tracking or individualized intervention. This simplification reflects a strategic cost–benefit evaluations by frontline staff rather than a lack of capacity (14). As one interviewee noted: “When there are too many patients, we just go through the motions. There’s really no energy to monitor everyone’s exercise routine” (FTDX_04). Such execution approximation represents a self-protective mechanism under limited resources and high pressure (26). While it may enhance short-term efficiency, it compromises the continuity and depth of interventions, ultimately deviating from the policy’s goal of service transformation.

In summary, the PHI policy has evolved along the Cognitive Pathway, from diverse interpretations to practical alienation. As the discourse descends through institutional layers, it becomes progressively simplified and re-translated, manifesting as goal redefinition, conceptual ambiguity, collaboration avoidance, and strategic compression. This cognitive deviation is a systemic outcome of institutional inertia, professional culture, and top-down policy diffusion. Understanding how the construction of policy cognition can help restore the link between understanding, collaboration, and action, while providing theoretical support for enhancing grassroots actors and improving policy operability.

### 4.2 Implementation dilemmas under resource and institutional constraints: the dual challenge of structural gaps and fragmented collaboration

While the PHI policy formally assigns expanded responsibilities and tasks to grassroots institutions, frontline implementers are widely constrained by both resource scarcity and institutional fragmentation. These constraints reflect longstanding systemic imbalances, leading to a disconnect, dilution, or even alienation of the policy’s original logic at the operational level (27).

At the resource level, primary healthcare actors face chronic shortages of personnel, limited budgets, and inadequate infrastructure, all of which hinder the institutionalization of exercise interventions (B1). As one township physician explained: “We’re already short-staffed as it is, and now we are being asked to do exercise interventions too. But there’s no dedicated personnel or space. Everything is arranged *ad hoc*” (FTDX_09). This concern was consistently observed across field sites, indicating that while the policy expands responsibilities, the corresponding human and material support has not kept pace. Consequently, implementation remains under-resourced despite comprehensive policy design (28). As Tummers et al. (29) note, frontline workers in high-pressure, low-resource contexts often resort to “passive compliance” or “selective implementation” to avoid systemic breakdowns.

Furthermore, imbalanced resource allocation exacerbates disparities in implementation capacity. Pilot areas with earmarked funding and policy privileges can establish dedicated intervention rooms and employ health coaches, whereas most non-pilot regions struggle to sustain such initiatives. This “concentration model of policy resources” is common in China and often produces what Zhu and Zhang (30) terms “demonstrative prosperity but systemic weakness”—success concentrated in showcase areas, with minimal system-wide improvement.

At the institutional level, segmentation and asymmetry further weaken collaborative capacity (B2). Although policy texts repeatedly emphasize “integration,” the sports and healthcare systems operate on parallel tracks in terms of data systems, performance evaluations, incentive structures, and governance logics. As one interviewee noted: “The health department has its own data system, and the sports bureau has theirs. They do not talk to each other. The assessments, budgets, and evaluations are all handled separately. There’s no real integration” (FTDX_05). This fragmentation leaves grassroots staff without joint directives or internal incentives for collaboration. In the absence of a cross-sectoral governance structure, complex policies risk falls into traps of coordination failure and governance fragmentation (31).

Finally, incentive mechanisms remain largely project-based and short-term, leading implementers to view exercise interventions as temporary assignments rather than long-term institutional commitments. As one practitioner observed: “Leaders require a report, some photos, and activity records every quarter. But whether residents stick to the plan or whether it’s actually effective—no one really asks about that” (FTDX_08). This reflects an evaluation system centered on superficial performance indicators, with limited attention to process quality or health outcomes. As Guo and Su (32) argues, such practices dampen motivation and undermine the depth of implementation.

Overall, resource scarcity and the absence of institutional coordination mechanisms constitute a fundamental dilemma in implementing the PHI policy. Confronted with a stark gap between high expectations and limited support, frontline actors increasingly adopt formalistic and defensive strategies. This not only weakens policy effectiveness but also obscures critical feedback mechanisms, delaying policy learning and revision cycles. Advancing genuine “integration” requires transcending sectoral silos and restructuring governance systems and resource allocation logics.

### 4.3 Differentiation of behavioral strategies: from institutional compliance to strategic adaptation

In implementing the PHI policy, grassroots healthcare workers are not passive recipients of top-down instructions. Under conditions of resource scarcity and institutional ambiguity, they develop diverse strategies that reveal both how the policy is enacted locally and the agency of frontline actors. These strategies can be grouped into three types: passive compliance, formalistic coping, and strategic adaptation.

In some grassroots institutions, healthcare workers adopt a passive compliance approach, responding only to direct oversight or performance pressure from higher authorities rather than proactively advancing the policy’s goals. As one interviewee noted: “After the residents complete their health check-ups, we organize a few exercise promotion talks. Beyond that, it’s hard to move things forward” (FTDX_10). This mode of implementation insufficiently advances the policy’s deeper goals but enables staff to meet performance evaluation requirements, representing a classic case of “compliance without commitment” (14). Cognitive deviations along Pathway Z1 lead many frontline staff to view the policy as a task imposed from above rather than as a meaningful long-term public health initiative. As a result, passive compliance becomes the default response.

Existing research indicates a lack of systematic evaluation mechanisms and incentive structures for PHI across China’s health and sports systems. The policy is often treated as a “soft task” rather than a mandatory objective, with few accountability or reward mechanisms to ensure effective implementation (33). Moreover, funding primarily comes from health and sports department budgets, with limited and uneven support from other sources (34). As one frontline worker observed: “Without any financial backing, health education is the cheapest way to meet the targets” (FTDX_01). This formalistic coping approach does not meaningfully advance the policy’s core objectives but reflects a pragmatic adaptation to resource constraints and institutional fragmentation. In the absence of adequate implementation tools and interdepartmental coordination, frontline actors gravitate toward low-cost, low-risk responses. While such practices may create the appearance of compliance, they often result in superficial execution, leaving grassroots health management systems unchanged. In some cases, this may even contribute to “illusory compliance” in the short term—seemingly meeting targets without delivering real public health outcomes (35).

Despite these constraints, some grassroots practitioners engage in proactive innovation, adapting implementation strategies to local conditions. For example, a community hospital in Yunnan Province partnered with a local senior university to develop a comprehensive health promotion platform: the hospital provided individualized exercise prescriptions, while the university’s instructors led the classes. Thus, they monitored and evaluated health outcomes over time.

This form of strategic adaptation goes beyond a mere response to resource scarcity, reflecting a deeper internalization of policy goals. Implementers view the policy not just as an administrative task but as a framework that can be adapted to local needs and capacities. Hence, they promote context-sensitive innovation, advance incremental progress toward national health objectives, and offer replicable models for other regions facing similar constraints.

### 4.4 Unintended consequences

In policy implementation, outcomes often extend beyond intended benefits to include a range of unintended consequences. These effects are not merely accidental but are closely linked to the implementation environment, as well as the management philosophies and operational modes adopted during execution (36).

The New Public Management (NPM) theory (37) emphasizes reducing bureaucratic rigidity and introducing market mechanisms to enhance the flexibility and efficiency of public service delivery. In PHI, some primary healthcare units have employed flexible management and innovative practices to advance policy goals. For example, cooperation between community hospitals and local senior universities demonstrates effective cross-sectoral collaboration, overcoming traditional governmental constraints and harnessing quasi-market mechanisms (36). Similarly, partnerships between community hospitals and local fitness organizations have facilitated low-cost exercise interventions, facilitating continuous data tracking, improving communication, and strengthening doctor-patient interactions. These cases illustrate how external resource mobilization and adaptive governance can enhance public service quality (38).

Conversely, in some grassroots medical institutions, the absence of long-term health intervention mechanisms and interdepartmental collaboration has led to symbolic responses, such as one-off health education sessions or perfunctory activities aimed at satisfying higher-level inspections. While these actions may achieve short-term objectives, they fail to produce meaningful health outcomes. From an NPM perspective, this reflects how frontline implementers in resource-constrained settings often rely on procedural and symbolic behaviors to meet performance targets, creating a disconnect between policy intention and actual impact (14).

Moreover, in the broader push for de-bureaucratization, unintended effects such as resource misalignment and implementation fatigue have emerged. Although NPM promotes de-bureaucratization to enhance governance agility, the lack of robust communication platforms and resource allocation mechanisms in PHI has increased, rather than alleviated, the burden on grassroots units.

Overall, the analysis demonstrates that the emergence of unintended consequences is a recursive feedback process, rather than a linear sequence. Specifically, deviations in policy cognition influence how frontline actors perceive available resources and institutional conditions, which in turn shape their behavioral strategies—ranging from passive compliance to adaptive innovation. The resulting outcomes—both positive and negative—feed back into the next cycle of policy interpretation and resource allocation, reinforcing or reshaping subsequent implementation patterns. This interactive loop forms a causal chain with feedback, linking cognition → resources → behavior → outcomes in a continuous and self-reinforcing process.

### 4.5 Strategies for policy improvement

The implementation of the PHI policy has revealed a chain of distortions—from cognitive biases and resource gaps to divergent behaviors and unintended consequences. This evolution reveals a recursive mechanism linking discourse translation, structural constraints, and differentiated execution strategies. Breaking this path dependency and transforming the policy from a “paper-based initiative” into an “institutionalized transformation” is now necessary. Based on the empirical findings, this study proposes the following strategies to support both policymakers and practitioners:

#### 4.5.1 Clarifying policy goals and task boundaries to strengthen cognitive ownership

This recommendation directly addresses findings under the Cognitive Pathway [Z1], where goal ambiguity and semantic drift led to misinterpretation of policy intent.

A core challenge lies in policy ambiguity. To counteract discourse alienation and target drift, the terminology and expectations of PHI need to be standardized. National authorities should issue authoritative guidelines, operational manuals, and case repositories to translate abstract concepts like “exercise intervention” and “integrated services” into actionable templates, reducing ambiguous interpretation and fragmented implementation.

Moreover, policy design should embed a co-production mechanism, involving frontline healthcare workers in early needs assessments and feedback sessions. This shift from “top-down” to “interactive governance” has been shown to enhance implementation motivation (39).

#### 4.5.2 Improving the resource and institutional support to build an “integrated organizational ecosystem”

Linked to Resource and Institutional constraints [Z2, Z3], this strategy responds to identified shortages of personnel, funding, and coordination mechanisms observed during axial coding.

The second challenge is capacity constraints, which are both operational and structural. To address the gap between expanding tasks and limited resources, the following system-level improvements are recommended:

1. Establishing a dedicated integration fund that incorporates exercise-based chronic disease interventions into the basic public health service subsidy system, with clearly defined fiscal safeguards and budget allocation mechanisms.
2. Institutionalizing joint taskforce meetings and checklist systems to delineate responsibilities between health and sports departments, coordinate workflows, and align budgetary frameworks.
3. Promoting shared information platforms to enable real-time data exchange between health records and exercise prescriptions. For example, Longhua District in Shenzhen established a “Medical-Sports Consortium,” where doctors prescribe individualized exercise plans, sports coaches deliver the sessions, and the platform tracks feedback, creating a closed-loop service system (34).

#### 4.5.3 Activating grassroots agency and encouraging positive strategic adaptation

Grounded in Behavioral Strategies [Z4] and Unintended Consequences [Z5], this approach builds on evidence of frontline innovation and policy adaptation identified through selective coding.

The third challenge is motivation and engagement. Strategic adaptations reveal the creativity of grassroots actors operating under bounded rationality and limited resources. Such innovations often arise in institutional environments that tolerate experimentation and moderate risk (29). Policy designers should avoid labeling all deviations from formal plans as “noncompliance,” and instead identify potential innovations embedded in frontline strategies.

To incentivize grassroots creativity, the following measures are proposed:

1. Launching awards such as a “Best Practice in Integration” Prize or “Innovation Pilot Program,” allowing localities to engage in controlled, exploratory experimentation.
2. Shifting performance evaluations toward process-based indicators, such as collaborative quality and service continuity, rather than short-term, symbolic outputs.
3. Offering capacity-building programs such as “Integration Action Coaching,” helping grassroots workers improve their cognitive understanding, technical skills, and collaborative competence.

PHI is a cross-system, cross-disciplinary policy experiment. Its challenges arise not from unclear objectives, but from the difficulty of embedding the policy within complex real-world settings. A comprehensive approach is needed to clarify cognition, supply resources, align mechanisms, and support action. Only by mobilizing the understanding, execution, and innovation capacities of grassroots actors can the vision of “integration on paper” be transformed into integration in practice.

## 5 Conclusion and discussion

### 5.1 Research findings

Drawing on the Grounded Theory approach, this study collected and analyzed 20 in-depth interviews with frontline medical practitioners at primary healthcare institutions in China. The aim was to investigate how grassroots implementers interpret, act upon, and generate differentiated outcomes during the implementation of the PHI Policy. A three-stage coding process and theoretical abstraction generate five major categories: policy cognition, resource and institutional constraints, behavioral strategies, policy translation mechanisms, and unintended consequences.

The findings reveal that grassroots implementation of the PHI Policy is not linear but a dynamic process of cognitive interpretation, strategic selection, and divergent outcomes. Abstract policy goals, limited institutional coordination, and uneven resource allocation led grassroots actors to adopt behavioral strategies such as passive compliance, symbolic responses, and strategic adaptation. These strategies produced highly context-specific implementation trajectories: some units achieved localized breakthroughs via intersectoral collaboration and resource innovation, while many others fell into performance mimicry and illusory compliance.

These findings highlight the complexity of policy implementation and illuminate the interplay between discourse reconstruction, resource constraints, and strategic adaptation. The study provides both a theoretical lens and practical insights for improving policy design and enhancing grassroots implementation capacity.

### 5.2 External validity and theoretical generalizability

Although the mechanism model of unintended consequences developed in this study is grounded in empirical data from China’s primary healthcare sector, its theoretical logic and operational dynamics demonstrate a level of generalizability and cross-context applicability.

First, in terms of policy typology, this model applies to other collaborative social policies, such as integrated medical-older population care, aging services, and coordinated community health–social service governance. These policies share common features: abstract policy goals, complex interdepartmental coordination, limited grassroots resources, and the coexistence of cognitive translation and behavioral divergence.

Second, regarding the institutional environment, the model can be extended to governance practices in other low- and middle-income countries (LMICs). Many face structural challenges—multi-level hierarchies, uneven resource distribution, and limited local policy absorption capacity—that often result in policy drift, symbolic implementation, or behavioral divergence in frontline execution (14, 31). By highlighting grassroots agency and strategic accommodation, this study offers a transferable lens for understanding local innovation under institutional constraints in LMIC contexts.

The model’s applicability depends on the institutional context. China’s centralized administration, performance-driven evaluation culture, and top-down governance heighten the risk of indicator-driven behavior and symbolic compliance. In more decentralized or incentive-diverse systems, such mechanisms may manifest differently. Therefore, while this model offers theoretical portability, its application requires contextual adaptation and local refinement.

Despite the robustness of the grounded theory approach, this study has certain limitations. The geographic coverage of participants was restricted to three regions (East, Central, and Southwest China), which may constrain the transferability of the findings. Moreover, the purposive sampling design and reliance on self-reported interview data may introduce subjectivity and potential response bias. Although this research applied triangulation, researcher cross-validation, and theoretical saturation tests to enhance credibility, future research should expand the regional scope and employ mixed-method approaches to strengthen external validity.

### 5.3 Research contributions

By focusing on the grassroots implementation of the PHI Policy, this study constructs a dynamic mechanism model that links policy cognition, behavioral strategies, and unintended consequences. It makes two significant contributions to the current literature:

#### 5.3.1 Enriching the conceptualization of grassroots agency in policy implementation studies

Traditional implementation theories often depict frontline actors as passive recipients in a top-down policy chain. In contrast, this study reveals that primary healthcare personnel act as active agents, making strategic choices shaped by cognitive framing and structural positioning. Their practices of strategic accommodation reflect a logic of selective innovation under institutional constraints, thereby extending the analytical scope of grassroots agency theory.

#### 5.3.2 Empirical refinement of new public management (NPM) assumptions in complex implementation settings

While NPM advocates performance orientation, decentralized governance, and cross-sectoral collaboration, this study finds that such principles often falter in practice due to institutional fragmentation, resource scarcity, and disjointed evaluation systems. Analyzing behaviors, like symbolic compliance and strategic adaptation, reveals a dual logic of superficial compliance and substantive deviation in resource-constrained, high-demand contexts. This provides an empirical test of Osborne’s (36) NPM framework and highlights the tensions between normative models and practical realities in complex governance systems.

## Data Availability

The original contributions presented in the study are included in the article/supplementary material, further inquiries can be directed to the corresponding author/s.
